# Respiratory co-infections in COVID-19-positive patients

**DOI:** 10.1186/s40001-023-01305-1

**Published:** 2023-09-02

**Authors:** Rania M Abd El-Halim, Hala Hafez, Ibrahim Albahet, Basma Sherif

**Affiliations:** 1 Clinical Pathology Department, Faculty of Medicine Ain Shams University, Cairo, 11566 Egypt; 2 Anaesthesia, Intensive Care and pain management department, Faculty of Medicine—Ain Shams University, Cairo, Egypt

**Keywords:** COVID-19, SARS-COV-2 PCR, Fungal co-infections, Galactomannan, Multidrug resistance, Secondary bacterial/fungal infections, Critically ill patients

## Abstract

**Background:**

Opportunistic respiratory infections may complicate critically ill patients with COVID-19. Early detection of co-infections helps to administrate the appropriate antimicrobial agent, to guard against patient deterioration. This study aimed at estimating co-infections in COVID-19-positive patients.

**Methods:**

Eighty-nine COVID-19-positive patients confirmed by SARS-COV-2 PCR were tested for post-COVID-19 lower respiratory tract co-infections through bacterial culture, fungal culture and galactomannan (GM) testing.

**Results:**

Fourteen patients showed positive coinfection with *Klebsiella*, nine with *Acinetobacter*, six with *Pseudomonas *and three with *E. coli*. As for fungal infections, nine showed coinfection with *Aspergillus*, two with *Zygomycetes* and four with *Candida*. Galactomannan was positive among one patient with *Aspergillus* coinfection, one with *Zygomycetes* coinfection and three with *Candida*, 13 samples with negative fungal culture were positive for GM. Ten samples showed positive fungal growth, however, GM test was negative.

**Conclusion:**

In our study, SARS-COV-2 respiratory coinfections were mainly implicated by bacterial pathogens; most commonly *Klebsiella* species (spp.), *Aspergillus* spp. were the most common cause of fungal coinfections, GM test showed low positive predictive value for fungal infection. Respiratory coinfections may complicate SARS-COV-2 probably due to the prolonged intensive care units (ICU) hospitalization, extensive empiric antimicrobial therapy, steroid therapy, mechanical ventilation during the COVID-19 outbreak. Antimicrobial stewardship programs are required so that antibiotics are prescribed judiciously according to the culture results.

## Introduction

COVID-19 disease has spread rapidly since 2019 and declared by WHO as a pandemic on March 2020. An enveloped novel coronavirus, (SARS-CoV-2), single-stranded RNA betacoronavirus of the family Coronaviridae, has arisen from Wuhan, China, in late 2019 which posed global healthcare and economic threats. Despite global containment and quarantine attempts, cases dramatically increased [1]. Although the majority of cases have asymptomatic or mild infections, significant proportion progress to severe pneumonia or acute respiratory distress syndrome (ARDS) which is associated with high mortality rates. Besides the impact of viral pneumonia itself, the prognosis can be affected by other factors as aging, ICU admission and infectious complications, such as bacterial or fungal infections [2]. Opportunistic infections following severe respiratory viral infections have been recognized since the 1918 influenza pandemic. Among critically ill patients with COVID-19, particularly secondary fungal infections caused by *Aspergillus*, *Candida* spp. and *Zygomycetes* are increasingly described [3]. Early detection of co-infections helps to administrate the appropriate antimicrobial agent, to guard against deterioration of the patient condition.

This study was conducted to determine susceptibility of COVID-19-positive patients to lower respiratory tract co-infections either bacterial or fungal.

## Patients and methods

### Specimen collection

From October 2021 to December 2021, 32,275 nasopharyngeal swab samples were submitted to the Molecular Microbiology Laboratory of Ain Shams University Hospitals to be tested for SARS-CoV-2 infection by real time-polymerase chain reaction (RT-PCR), from which 89 positive SARS-CoV-2 samples were chosen, where those patients required hospital admission as they were classified as severe cases with acute respiratory illness according to WHO COVID-19 Case definition [4] and also combined bacterial and fungal sputum cultures were ordered to exclude lower respiratory tract coinfection. Nasopharyngeal swab samples were placed in viral transport media (VTM) (disposable virus sampling swab kits, Bioteke corporation, Wuxi, Co., Ltd., China) for RNA extraction. Sputum cultures, both bacterial and fungal, were performed for COVID-19 PCR-positive patients. After that, sputum specimens were stored at − 70 °C to perform galactomannan assay.

## Sample processing

### SARS-COV-2 detection by RT-PCR on nasopharyngeal swabs

SARS-CoV-2 RNA extraction was performed using Chemagic™ Viral DNA/RNA 300 H96 magnetic bead-based Kit utilizing chemagic™ 360 Nucleic Acid Extractor (PerkinElmer, USA), followed by RT-PCR using VIASURE SARS-CoV-2 RT-PCR Detection Kit (CerTest Biotec SL, Spain) to detect SARS-CoV-2 specific genes; Orf and N genes in nasopharyngeal swabs. The reverse transcription and amplification was performed in the Bio-Rad CFX 96 System (Bio-Rad Laboratories, Inc, USA) according to the following program: one cycle of reverse transcription at 45 °C for 15 min, Forty cycles of both denaturation at 95 °C and annealing, extension as well as fluorescence acquisition at 60 °C. RNA was extracted from respiratory specimens, reverse transcription and the subsequent amplification of a conserved region of ORF1ab and N genes for SARS-CoV-2 occurred in the same reaction well using specific primers and detected using fluorescent reporter dye probes. Virus concentrations in samples were estimated from cycle threshold (Ct) value [5].

### Sputum specimen collection

Specimens were collected under the guidance of a well-trained healthcare personnel to inform the patient about proper collection technique including rinsing the mouth with water followed by expectorating deep cough sputum directly into a sterile, leak-proof, screw-cap collection container. Only high-quality sputum samples as defined by Bartlett^'^s criteria were included is the study [6].

### Bacterial culture of sputum specimens

Direct Gram staining of sputum samples was performed, examined and reported in the form of Q-scoring. Each sputum specimen was cultured on Blood agar, Chocolate agar and MacConkey agar plates (Oxoid, UK) by quadrant technique, plates were incubated aerobically for 24–48 h at 37 °C. Positive bacterial cultures were identified through manual identification using Gram stain, colony morphology, biochemical reactions and API (BioMérieux, France). Antibiotic susceptibility testing was conducted on positive bacterial cultures by Kirby–Bauer disc diffusion method and interpreted according to Clinical Laboratory Standard Institute [7], discs were purchased from (Oxoid, UK).

### Fungal culture of sputum specimens

Each specimen was cultured on two Sabouraud Dextrose agar (SDA) plates (Oxoid, UK) supplemented with chloramphenicol according to the manufacturer’s instructions in a concentration of 100 mg/L, one plate was incubated aerobically at 28 °C, the other one at 37 °C and regularly checked for growth every 2 days for 2 weeks. Positive fungal cultures were identified through colony morphology and methylene blue stained film.

### Galactomannan assay performed on sputum specimens

Specimens were completely thawed, homogenized, thoroughly vortexed, then centrifuged for 20 min at 2000 rpm. Supernatant was removed and assayed immediately according to manufacturer instructions (SinoGeneClon, Biotech Co.), absorbance was read on a spectrophotometer at 450 nm OD. OD index ≥ 0.5 was interpreted as a positive result as suggested by the FDA [8].

### Statistical analysis

The collected data were revised, coded, tabulated and introduced to a PC using Statistical Package for Social Science (SPSS 20). Data were presented and suitable analysis was done according to the type of data obtained for each parameter.

## Results

Eighty-nine COVID-19-positive patients confirmed by RT-PCR were tested for post COVID-19 lower respiratory tract co-infections through bacterial culture, fungal culture and GM testing. Bacterial culture showed 32 (35.9%) positive samples, where, 14 (15.7%), 9 (10.1%), 6 (6.7%), 2 (2.2%) and 1 (1.1%) showed positive coinfection with *Klebsiella*, *Acinetobacter*, *Pseudomonas*,

*Escherichia coli (E. coli)* and *Stenotrophomonas* respectively (Table 1). Fungal culture showed 15 (16.9%) positive samples, where 9 (10.1%), 2 (2.2%) and 4 (4.5%) showed positive coinfection with *Aspergillus*, *Zygomycetes* and *Candida* respectively. Five samples with positive fungal growth were positive for GM (33.3%) Table (1), 74 samples were negative for fungal growth, of which, GM was positive in 13 samples (17.6%). Fifteen samples were positive for fungal growth, of which, GM was negative in 10 samples (66.7%).

**Table 1:** Percentage of fungal/bacterial co-infections recovered from PCR-positive COVID-19 patients

	SARS-COV-2 coinfection	Percentage (%)	Positive GM	Percentage/fungal species (%)	Percentage/total fungal growth (%)
Fungal species					
* Aspergillus*	9	10.1	1	11.1	6.7
* Candida*	4	4.5	3	75	20
* Zygomycetes*	2	2.2	1	50	6.7
Total	15	16.8	5		33.4
Bacterial species					
* Klebsiella*	14	15.7			
* Acinetobacter*	9	10.1			
* Pseudomonas*	6	6.7			
* E. coli*	2	2.2			
* Stenotrophomonas*	1	1.1			
Total	32	35.9			

## Discussion

Respiratory viral infections predispose to secondary infections, whether bacterial or fungal, where, viral infection of the lungs dampens the immune system, thus results in alterations in the population of respiratory microbiota. Co-infections usually carry a worse prognosis, especially in patients with comorbid conditions, such as diabetes, hypertension or coagulopathies. Multiple studies have documented a higher risk clinical status or increased mortality rate. The extensive use of antibiotics during the early stage of the pandemic might have contributed to the development of the increased risk of bacterial or fungal superinfection [9] as well as the development of resistant strains. Bacterial or fungal coinfection negatively affect the outcomes of COVID-19 patients due to the aggressive synergism, besides, bacterial superinfection may exaggerate the hyperinflammatory status, leading to a cytokine storm [10]. Thus, the presence of a coinfection should be meticulously investigated during the diagnosis of COVID-19 to provide early proper management [9]*.*

In our study, 89 COVID-19-positive patients confirmed by SARS-COV-2 PCR were tested for post-COVID-19 lower respiratory tract co-infections through bacterial culture, fungal culture and GM testing. Forty-seven patients (52.8%) showed positive COVID 19 coinfection; 32(36%) were caused by bacterial pathogens and 15 (16.9%) were caused by fungal ones. This is concordant with Chong et al. [11], where, 16% of secondary infections were caused by bacterial infections and 6.3% caused by fungal infections of COVID-19 patients. Most bacterial coinfections were caused by multidrug resistant (MDR) strains, this is concordant with Polly et al. [12] who reported that 29.7% of COVID-19 associated infections were implicated by bacterial MDR, this may be attributed to multiple factors, e.g., mechanical ventilation, immune dysregulation, administration of prophylactic antimicrobials as well as immunomodulatory therapies, such as corticosteroids and IL-6 inhibitors. The great pressure on the healthcare facilities due to the rapid spread of the pandemic together with the worldwide shortage in the disinfecting agents and the personal protective equipment, all these factors together may have impeded the proper optimum patient care or predisposed to coinfection with multidrug resistant strains. Multidrug resistant *Klebsiella* species were the most common organism implicated in this coinfection among 14 (15.7%) cases, followed by other Enterobacterales and non-fermenting Gram-negative bacilli (Table 1). This was concordant with Simmonds et al. [13] who identified 31 carbapenemase-producing Enterobacterales isolates from positive COVID 19 patients, including 27 *Klebsiella pneumoniae* (*Kl. pneumoniae*). Also, Pasero et al. [14] reported that the bacterial MDR infections secondary to COVID-19 was between 30 and 50%, where, *Pseudomonas aeruginosa (P. aeruginosa)*, *Staphylococcus aureus (S. aureus)*, *Kl. pneumoniae*, methicillin-resistant *S. aureus* (MRSA) and carbapenem-resistant Gram-negative bacteria represented 35%, 23%, 19%, 10% and 32%, respectively. Chong et al. [11] reported that the most frequent bacterial agents isolated from the respiratory tract were* P. aeruginosa, Klebsiella* spp., *S. aureus, E. coli* and *Stenotrophomonas maltophilia* representing 21.1%, 17.2%, 13.5%, 10.4%, and 3.1%, respectively.

However, Feldman and Anderson 2021 [15] stated that *Streptococcus pneumoniae*, *Moraxella catarrhalis*, *S. aureus* and *Haemophilus influenzae* were the most common bacterial pathogens implicated in COVID-19 coinfection. It is worth mentioning that using antibiotics either empirically or based on culture and susceptibility test for COVID-19 bacterial coinfection may predispose to fungal infections [16]. In our study, 15 (16.9%) samples showed positive fungal growth, where 9 (10.1%), 2 (2.2%)and 4(4.5%) samples showed positive coinfection with *Aspergillus*, *Zygomycetes* and *Candida* respectively. 1/9 of *Aspergillus*, 1/2 of *Zygomycetes* and 3/4 of *Candida*-positive cultures showed positive GM results (Table 1). This was concordant with Segrelles-Calvo et al. [17], who reported 5.4% cases with pulmonary aspergillosis among positive COVID-19 patients. However, Hughes et al. [18], reported that *Candida* spp. isolates were the most common (21.4%), however, these isolates were probably oropharyngeal thrush or normal flora rather than pulmonary candidiasis. Also, *Aspergillus fumigatus* were identified in 2.7% of cases. Ten samples (66.7%) showed positive fungal growth (eight samples of which were positive for *Aspergillus*), however were GM negative.

Similar findings were reported by Alanio et al. [19], where serum GM was negative in eight of nine (89%) patients who had positive *Aspergillus* coinfection, suggesting a lesser degree of *Aspergillus* invasiveness or early invasive pulmonary aspergillosis. Also, Verweij et al. [20], stated that only three (21%) of 14 patients with COVID-19-associated pulmonary aspergillosis were serum GM positive. Similarly Melancon et al. [21] reported that, overall GM sensitivity was 44.8%, and Hsu et al. [22], mentioned that, the use of mold-active antifungal prophylaxis decreased the overall sensitivity of the BAL GM; where, sensitivity was 76% in those who received prophylaxis versus 91% in those who did not receive it. Other factors that may lower GM sensitivity include the rate and extent of secretion from the fungus and the rate of GM elimination [22].

Seventy-four samples were negative for fungal growth, among which, GM was positive in 13 samples (17.6%) which may be attributed failure of growth of some fungal spp. on SDA or false GM positivity caused by cross-reactivity with antibiotics, e.g., B-lactams (piperacillin/tazobactam or amoxicillin/clavulanate), blood products, elevated IgG levels or intravenous immunoglobulin (IVIG) administration [23].

It is worth mentioning that the fate of coinfected patients was aggressive in our study, where 10 patients were admitted to the ICU, with regard to oxygen therapy; three patients required oxygen mask, six patients required Venturi 50% Nasal 2 L, and nine were on mechanical ventilation and three patients died. The ICU patients were coinfected by MDR strains of *Klebsiella*, *Acinetobacter*, *Pseudomonas or Aspergillus*. The dead patients suffered *either Zygomycetes* or MDR *Klebsiella* coinfection. Other patients with non-MDR bacterial co-infections or *Candida* co-infections received the appropriate antimicrobial therapy in their ward. All patients were discharged after completion of the antimicrobial course and improvement of clinical status upon the consultant approval.

In conclusion, SARS-CoV-2 bacterial or fungal coinfection represents a serious problem for the healthcare facilities worldwide where it increases the morbidity and mortality rates as well as the healthcare cost. Diagnosis of coinfection is challenging; clinical symptoms are often nonspecific thus clinical diagnosis is sometimes difficult. Bacterial and fungal sputum cultures testing for suspected patients allow early detection and management of coinfection. The GM test is simple, widely available, standardized and objective however, the test has some drawbacks because, GM concentration in vivo is determined by the rate of production and secretion by the growing fungus. Also, it has limited range of detection of fungal species mainly *Aspergillus* species. Owing to the low positive predictive value, we recommend Beta-D-glucan (BDG) to be tested due to the fact that BDG is more sensitive and covers a broad spectrum of fungal species.

Most bacterial coinfections are caused by multidrug resistant strains, thus, surveillance and antimicrobial stewardship programs, in addition to provision of alternative treatment approaches are ultimate needs to mitigate their upcoming effect. In case of fever refractory to antibiotic treatment, fungal infection should be suspected and investigated for early administration of antifungal agents to reduce morbidity and mortality.

## Data Availability

The authors declare that data supporting the findings of this study are available within the article and its additional files**.**

## Abbreviations

SARS-CoV-2: Severe acute respiratory syndrome coronavirus 2
COVID-19: Coronavirus disease 2019
VTM: Viral transport media
RT-PCR: Real time-polymerase chain reaction
GM: Galactomannan
ICU: Intensive care units
ARDS: Acute respiratory distress syndrome
SDA: Sabouraud dextrose agar
SPSS: Statistical Package for Social Science
MDR: Multidrug resistant
*E. coli*: *Escherichia coli*
*Kl. pneumoniae*: *Klebsiella pneumoniae*
*P. aeruginosa*: *Pseudomonas aeruginosa*
*S. aureus*: *Staphylococcus aureus*
MRSA: Methicillin-resistant *S. aureus*
IVIG: Intravenous immunoglobulin
BDG: Beta-d-glucan
